# Evaluating early implementation of the innovative Canadian policy of cigarette stick warnings among adults in Canada who smoke: An assessment using repeat cross-sectional surveys and daily diaries

**DOI:** 10.18332/tid/211649

**Published:** 2025-11-26

**Authors:** Emily E. Hackworth, Yanwen Sun, Samantha Petillo, Liyan Xiong, Dèsirée Vidaña-Pérez, Chih-Hsiang Yang, Minji Kim, Crawford Moodie, Stuart Ferguson, David Hammond, Jeff Niederdeppe, James F. Thrasher

**Affiliations:** 1 Masonic Cancer Center, University of Minnesota, Minneapolis, United States; 2 Department of Health Promotion, Education and Behavior, University of South Carolina, Columbia, United States; 3 Department of Biostatistics and Epidemiology, University of South Carolina, Columbia, United States; 4 Department of Exercise Science, University of South Carolina, Columbia, United States; 5 Institute for Social Marketing and Health, University of Stirling, Stirling, United Kingdom; 6 Tasmanian School of Medicine, University of Tasmania, Hobart, Australia; 7 School of Public Health Sciences, University of Waterloo, Ontario, Canada; 8 Department of Communication and Jeb E. Brooks School of Public Policy, Cornell University, Ithaca, United States

**Keywords:** smoking, cessation, global health, FCTC

## Abstract

**INTRODUCTION:**

In 2024, Canada became the first country to implement warning messages on cigarette sticks. Warnings were required on king-size cigarettes in April 2024 at the manufacturer level and July 2024 at the retail level. The purpose of this study was to evaluate responses to cigarette stick warnings among adults who smoke in Canada using a standard survey and a daily diary study.

**METHODS:**

We used two separate online survey (i.e. questionnaire) methods with Canadian adults who smoke daily and use king-size cigarettes, with data collected in February, May, and August 2024. The first method was a standard cohort survey (observations=1724; participants=999), with one survey each data collection period. Participants were followed up in subsequent waves. Participants reported noticing health information on cigarette sticks ‘any’ vs ‘none’, and ≥ ‘almost all’ vs ‘fewer cigarettes’ in last month. The second method was a daily diary study (observations=10572; participants=527), with brief surveys every evening for two weeks during each data collection period. Participants reported noticing health information on cigarette sticks (‘any’ vs ‘none’ in last 24 hours). Samples for the two studies were distinct. In both studies, we also assessed feelings about the look of cigarette sticks (1=very bad to 5=very good), forgoing cigarettes normally smoked (no vs yes), and quit motivation (continuous). Generalized estimating equations regressed outcomes on survey period, adjusting for sociodemographic and smoking-related covariates.

**RESULTS:**

Noticing stick warnings increased in both surveys [standard ‘any’: May=58%, August=73%, OR=2.29 (95% CI: 1.81–2.91); standard ≥ ‘almost all’: May=27%, August=44%, OR=2.56 (95% CI: 1.99–3.30); daily diary: February=6%, May=10%, OR=1.77 (95% CI: 1.29–2.44), August=16%, OR=2.92 (95% CI: 1.73–4.93), all p<0.001]. Over time, negative feelings toward sticks [February=4.10, August=3.91, mean diff= -0.19 (95% CI: -0.32 – -0.05), p=0.006], forgoing cigarettes [February=56%, August=63%, OR=1.44 (95% CI: 1.12–1.86), p=0.004] and quit motivation [February=4.74, August=5.03, mean diff=0.30 (95% CI: 0.06–0.53), p=0.014] increased in the standard surveys, but not the daily diary study.

**CONCLUSIONS:**

Canadian adults who smoke king-size cigarettes increasingly noticed cigarette stick warnings over the early implementation period. The standard survey also found increases in cessation-related responses to stick warnings. Future research should assess long-term impacts of this policy and validate standard and daily diary survey methods for evaluating labeling policies.

## INTRODUCTION

Cigarette smoking remains a leading preventable cause of premature death and disease worldwide^1^. To communicate health risks from smoking to the public, most countries require warnings on cigarette packs, with warnings increasingly covering more than half the primary pack display areas and including images to illustrate text warning statements^2^. The WHO Framework Convention on Tobacco Control (FCTC) has played a pivotal role in the adoption of prominent pictorial warnings^3^, explicitly recommending those that cover at least half of the pack and providing a medium for sharing health information^4^. Strong warnings on packs can increase knowledge of smoking risks, make cigarettes less appealing, and reduce smoking rates^3^. However, the effects of warnings, irrespective of their size and content, wear out over time, as consumers are repeatedly exposed to the messages^5^.

Canada has strong tobacco labeling policies, being the first country to implement pictorial warnings on cigarette packs in 2001^6^. In 2012, Canada updated the content of its 16 rotating pictorial warnings, and increased the warning size from 50% to 75% on the front and back of cigarette packages^2^. Standardized (or plain) packaging was implemented in 2020^7^ and, in 2024, Canada again implemented new content for pictorial warnings and became the first country to require warnings printed on cigarette sticks^8^. Cigarette stick warnings were required first on all king-size cigarettes sold after July 2024, while for regular-size cigarettes the start date was end of April 2025^8^.

Printing warnings on cigarette sticks extends health messaging beyond the package and into the smoking session^6^. Cigarette packaging is not necessarily visible at the point of consumption, while on-cigarette warnings could be viewed after the pack is opened and the consumer pulls the cigarette out of the pack, handles the cigarette while smoking, when a cigarette is in an ashtray, and even after it is put out^4^. There is a growing body of research in support of on-cigarette warnings^9^, including qualitative research. For instance, focus group participants in Scotland reported that on-cigarette warnings could serve as a constant reminder of the health risks of smoking^4^ and encourage those who smoke to stub out cigarettes early, reduce consumption, or motivate them to quit^10^. Stubbing out and cutting back on cigarettes are possible precursors to cessation among those who smoke^11^. A review of qualitative and experimental research on the impacts of dissuasive cigarettes, including studies from Australia, New Zealand, the United Kingdom (UK) and Norway, supports the implementation of on-cigarette warnings, finding that they were associated with reduced appeal, increased perceptions of harm, lower trial intentions and increased intentions to quit smoking when compared to cigarettes without warnings^12^. These findings are supported by a study of adults who smoke in Canada, finding that negative affect about cigarette sticks and forgoing behaviors increased after the policy was implemented, both of which were associated with subsequent attempts to quit smoking^13^. However, this study included people who smoked regular-size cigarettes (which did not include warnings) and compared pre and post policy periods without assessing the roll-out of the policy.

In June 2023, Health Canada announced its updated labeling policy, including the phased introduction of on-cigarette warning messages for all king-size cigarette sticks, which had to include one of six brief rotating messages printed on the filter paper by the end of July 2024 (Supplemental file Figure 1)^8^. In January 2024, warnings began appearing on king-size sticks – which accounted for approximately 70% of legal cigarette sales in Canada^14^.

The current study used two survey methods to evaluate responses to cigarette stick warnings among adults in Canada who smoke king-size cigarettes, each assessing trends across the early policy implementation period, covering the period immediately before and after the manufacturer and retail sales deadlines. We hypothesized that both survey methods would demonstrate increases in exposure to on-cigarette warnings over this early implementation period, with additional increases in cessation-related psychosocial and behavioral outcomes that may be related to these exposures.

## METHODS

### Procedure and participants

We analyzed data from two concurrent surveys, both involving questionnaires administered during three moments in time (February 2024, May 2024, August 2024) over which warnings were implemented on king-size cigarettes. The first survey method comprised a standard survey every 3 months (hereafter, standard survey), as part of a larger open cohort study to assess the population-level effects of the updated Canadian warning label policy. The second method was a 2-week daily diary study that was repeated every 3 months (hereafter, daily diary study), to allow for a fine-grained assessment of responses to labeling using questions with shorter recall timeframes. For each study, survey data were pooled to permit assessment of differences in outcomes across each of the three survey periods. For both studies, ethics approval was obtained from the University of South Carolina Institutional Review Board. All participants provided informed consent prior to initial enrollment and at each subsequent wave of participation.

*Standard survey*

Every three months from February 2023 to February 2025 we surveyed approximately 1500 adults in Canada who smoked cigarettes. Participants were recruited via an online panel provider (Leger), with the following eligibility criteria: being aged ≥18 years, reporting at least ≥100 lifetime cigarettes, having smoked at least once in the prior month, and being able to read English and/or French. Respondents completed the survey in their preferred language.

Following initial recruitment, participants were re-recruited for the subsequent survey, regardless of whether they continued to smoke or not, achieving an average retention rate of 70% (range: 62–73). To maintain a consistent sample size of approximately 1500 participants per wave, the sample was replenished with newly recruited adults who smoked. Soft quotas were implemented to ensure demographic representativeness across key characteristics (age, sex, education level, and provincial residence), aligned with the general Canadian adult population.

To align this sample with the daily diary study inclusion criteria, the present analysis was limited to participants in the February 2024, May 2024, and August 2024 survey waves. Furthermore, we used the same inclusion criteria as for the daily diary study, including only those who smoked daily, used factory-made cigarettes at least as frequently as roll-your-own cigarettes, and who did not use e-cigarette products in the past 30 days at the time of the survey, the latter of which was done to avoid complexities around analyzing switching and compensatory behaviors across multiple products (n=2377 observations from 1388 individuals). Given that the policy was initially implemented just for king-size cigarettes (i.e. 84 mm long), we excluded participants who did not usually use king-size cigarettes (n=881 or 29.1%), resulting in a final analytic sample of 1724 observations from 999 participants (February 2024, n=573 participants; May 2024, n=555 participants; August 2024, n=596 participants).

For the standard survey, most variables had <5% missing data, except ‘noticing smoking harm information on cigarette sticks’, which had about 33% missingness.

*Daily diary study*

The daily diary study involved a two-week period of daily surveys (at the end of each day) across the same three survey periods as the standard survey (February, May, and August 2024). Participants were recruited from the same Canadian online panel provider as the standard survey to ensure that nobody participated in both surveys during the same survey period. Eligibility criterion for the daily diary study varied slightly. Participants were eligible if they reported smoking >100 cigarettes in their lifetime, were daily smokers, had not vaped in the past 30-days, and smoked mostly factory-made cigarettes.

At each survey wave, approximately 600 participants were screened as eligible and completed an initial baseline assessment. As in the standard survey, previously enrolled participants were invited to participate in subsequent waves, supplemented by new recruits to maintain the target sample size. Retention rates were 41% between February and May 2024, and 51% between May and August 2024. About one-fifth (19%) of participants completed all three waves.

After baseline screening and assessment, an average of 89% of participants across the three waves of data collection consented to the 14-day daily diary study. Each day, participants received standardized email communications at 19:00 local time, inviting them to complete a brief survey of 2 to 4 minutes. The current analysis incorporated data from participants who completed at least 11 of the 14 daily diaries (n of observations=14112 or 79.2%) to ensure completeness of data. As with the standard survey analysis, we excluded participants who did not use king-size cigarettes (n of observations=463 or 23.2%), generating an analytical sample with 10572 observations from 527 participants (February 2024, n=2943 observations from 226 participants; May 2024, n=4162 observations from 321 participants; August 2024, n=3467 observations from 268 participants). In the daily diary survey, missingness was <5% for all variables.

### Measurement

Four constructs were assessed in both the standard survey and daily diary study: 1) noticing cigarette stick warnings, 2) affective responses to cigarette sticks, 3) forgoing any cigarettes, and 4) motivation to quit smoking. All questions were adapted from previously validated instruments. We conducted cognitive interviews with 10 Canadian adults who smoke prior to the initial wave deployment to confirm understanding of our measurement instructions to consider their cigarette sticks only, as well as question wording.

*Noticing warnings on cigarette sticks*

In the standard survey, participants were asked: ‘In the last 30 days, how many of the cigarettes you smoked had health messages on the cigarette stick paper?’. Responses were dichotomized using two distinct approaches: none=0 vs any=1; and none/a few/some/about half=0 and almost all/all=1. This item was administered only in the May and August 2024 surveys (as we did not find out until after our February survey that on-stick warnings were circulating by January 2024). In the daily diary study – which included data collection in February 2024, before policy implementation deadlines – the question used aimed to minimize potential attribution bias and demand effects that our repeated, daily assessments could have caused. Hence, daily diary participants were asked ‘Today, did you notice any information about harms from smoking?’ with those who provided affirmative responses subsequently asked: ‘Where did you notice information today about harms from smoking?’. Response options encompassed online (websites, social media), print media (newspapers, magazines, posters), television, cigarette packages, and cigarette sticks. Participants selecting cigarette sticks were coded as 1; all others, including those not reporting noticing any such information, were coded as 0. This approach ensured that participants were repeatedly prompted to think about anything specific to the cigarette stick warning before the policy was implemented.

*Affective responses to cigarette sticks*

In both surveys, participants were prefaced with the statement: ‘We are interested in knowing what you think about the cigarette sticks you smoke – not the package the cigarettes come in’. The standard survey assessed emotional responses with the item: ‘How do you usually feel when you look at the cigarette sticks you smoke?’ (1=very bad to 7=very good). The daily diary study incorporated a comparable item: ‘Today, how did you usually feel when you looked at the cigarette sticks you smoke?’ (1=very bad to 7=very good; with an additional option: did not look at my cigarette sticks). Both items were based on previously validated items^15^.

*Forgoing cigarettes*

The standard survey assessed past-month cigarette forgoing with the item: ‘In the past 30 days, how often, if at all, have you stopped yourself from having a cigarette when you had the urge to smoke?’ (recoded as 0=none, 1=any). In the daily diary study, same-day cigarette forgoing was measured by: ‘Today, did you choose to skip any cigarettes that you normally would have smoked?’ (recoded as 0=none, 1=any). Similar items on pack warning responses from which these were adapted showed sound measurement properties^16^.

*Motivation to quit*

In the standard survey, quit motivation was assessed with the item: ‘How motivated are you to quit smoking?’ (1=not at all to 10=extremely)^17^. This measure was adapted for the daily diary study, in which participants reported their same-day motivation to quit: ‘Today, how motivated have you been to quit smoking?’ (1=not at all to 5=extremely).

*Covariates*

Participants reported their biological sex at birth, age (recoded to 0=18–34; 1=35–49; 2=50–64; 3= ≥65 years), education level (recoded to: 0=high school or lower; 1=technical school; 2=college or higher), and race/ethnicity (recoded to 0=White; 1=other). Smoking frequency was recoded based on cigarettes smoked per day (recoded to 0=0–9; 1=10–14; 2=15–19; 3= ≥20). Participants also reported past month use of roll-your-own tobacco (recoded as 0=none; 1=any), as warnings were not mandatory for rolling papers, as well as past month use of cigarettes purchased from First Nations Reserves (recoded to 0=none; 1=any), where compliance with labeling requirements is challenging to enforce. Participants also reported their intentions to quit smoking (recoded to 0=intend to quit after six months, no intention, or don’t know; 1=within the next month, three months, or six months) and any quit attempt in the prior 3 months (recoded to 0=none; 1=any).

### Statistical analysis

We estimated descriptive statistics for participants in each study, comparing their characteristics using chi-squared tests. To examine temporal trends across the four outcome variables in both surveys, we employed generalized estimating equations (GEE) to account for within-subject correlation from repeated measurements (i.e. standard errors clustered at the individual level). An exchangeable correlation structure with robust standard errors was specified in all models. We excluded participants who selected ‘don’t know’ or ‘refused’ for outcome variables in the standard survey [i.e. noticing: n=69 (4.78%); feeling about cigarette sticks: n=79 (3.67%); forgoing: n=26 (1.51%); motivation to quit: n=26 (1.21%)]. We excluded observations in the daily diary study where participants responded: ‘Did not look at my cigarette sticks’ when analyzing feelings toward cigarette sticks (n=2146 or 20.3%). Linear GEE models were utilized for continuous outcomes (motivation to quit, thinking about harms, and negative feeling toward cigarette sticks), while logistic GEE models were employed for binary outcomes (any forgoing, noticing any cigarette stick warnings, noticing almost all cigarette sticks having warnings). Across models, the main independent variable was survey wave (February=reference group), and all models were adjusted for covariates mentioned above as well as time-in-sample (i.e. number of previous survey waves completed at the time of the survey period).

Post-estimation margins were computed to derive adjusted means and proportions for each wave, with figures generated to show trends over time. The graphs were derived from margins estimates and 95% CIs obtained from the GEE regression models; we then plotted these values across waves. In the daily diary study, we calculated intraclass correlation coefficients (ICCs) representing average agreement across observations within participants, following multilevel mixed-effects logistic regression models with participant-level random effects, to assess the proportion of variance attributable to between-person differences over time. As sensitivity analyses, we then re-ran all models after including all daily observations (including those from people who completed less than 11 daily diaries). In additional sensitivity analyses, post-stratification weights were applied to the standard survey each wave to adjust for the sex, age, and education distribution of Canadian adult smokers, based on the 2021 Canadian Community Health Survey. All analyses were conducted using Stata 18.0, with two-tailed tests and statistical significance set at p<0.05.

## RESULTS

The demographic and behavioral characteristics of participants in the standard and daily diary studies are presented in Table 1. The samples were similar in the distribution of sex, race, cigarettes per day, purchasing cigarettes from First Nation reserves, quit intention, and use of roll-your-own tobacco. Compared to the daily diary study, the standard survey included a higher proportion of participants aged 18–34 years (standard=14%, daily diary=6%, p<0.001), those with a high school education or lower (standard=50%, daily diary=25%, p<0.001), and individuals who reported a quit attempt in the past three months (standard=27%, daily diary=22%, p<0.001). Additionally, the distribution of participants across waves was more balanced in the standard survey, whereas there were fewer respondents in the wave 1 daily diary study compared to later waves.

**Table 1:** Characteristics of two samples of Canadian adults who smoke king-size cigarettes, surveyed in February, May, and August 2024

*Characteristics*	*Categories*	*Standard survey ^a^*		*Daily diary study ^b^*
		*Unweighted %*	*Weighted %*	*Unweighted %*
**Age** (years)	18–34	14	12	6***
	35–49	23	23	24
	50–64	41	42	45**
	≥65	22	23	25*
**Sex**	Female	54	49	58**
	Male	46	51	42**
**Race**	White	86	87	85
	Other	14	13	15
**Education level**	High school or lower	50	51	25***
	Technical school	36	38	42***
	College or higher	14	11	32***
**Cigarettes per day**	0–9	23	22	25
	10–14	24	24	27*
	15–19	17	17	18
	≥20	36	37	31***
**First nation**	Any	38	37	40
**Quit intention**	In next 6 months	31	31	29
**Roll-your-own use**	Any in last 30 days	12	11	10
**Quit attempt**	Any in last 3 months	27	25	22***
**Survey wave**	Feb 2024	33	33	28***
	May 2024	32	32	39***
	Aug 2024	35	35	33
**Number of surveys completed**	1	31	31	38***
	2	23	23	33***
	3	46	46	29***

a N=1724 observations in the table from 999 individuals.

b N=10572 observations shown in the table from 527 individuals.

* p<0.05.

** p<0.01.

*** p<0.001.

### Temporal trends

**Figure 1:**
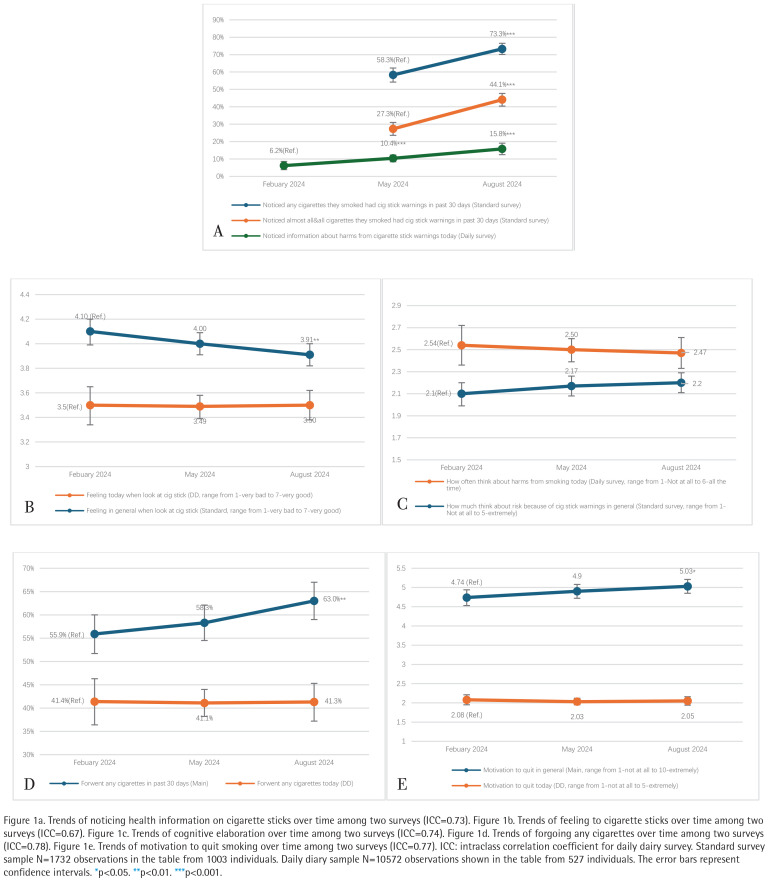
Different trends of response variables among Canadian adults who smoke king-size cigarettes in different surveys, February –August 2024

Figure 1 illustrates differential temporal trends in response variables. The frequency of noticing cigarette sticks increased over time for both survey methods (all p<0.001). More participants in the standard survey reported that they had noticed health information on cigarette sticks in August compared to May, whether for noticing this information on ‘any’ cigarette sticks [May=58.3%, August=73.3%, OR=2.29 (95% CI: 1.81–2.91), p=0.0000] or on almost all or all cigarette sticks they smoked [May=27.3%, August=44.1%, OR=2.56 (95% CI: 1.99–3.30), p=0.0000]. In the daily diary study, the proportion of smokers who noticed smoking harm information on cigarette sticks that day was significantly higher in both May [10.4%, OR=1.77 (95% CI: 1.29–2.44), p=0.0005] and August [15.8%, OR=2.92 (95% CI: 1.73–4.93), p=0.0006] compared to February (6.2%, p<0.001). No other statistically significant changes over time were observed in the daily diary survey. By contrast, in the standard survey, feeling about cigarette sticks was more negative in August (mean=3.91, SD=0.05) than February [mean=4.10, SD=0.05, mean diff= -0.19 (95% CI: -0.32 – -0.05), p=0.006]; forgoing cigarettes was higher in August (63.0%) than February [55.9%, OR=1.44 (95% CI: 1.12–1.86), p=0.004]; and motivation to quit smoking was higher in August (mean=5.03, SD=0.09) than February [mean=4.74, SD=0.10, mean diff=0.30 (95% CI: 0.06–0.53), p=0.014]. Cognitive elaboration did not change significantly over time in both standard and daily diary studies (range of mean in the standard survey=2.1–2.2; range of mean in the daily diary survey=2.47–2.54).

**Table 2:** Correlates of noticing cigarette stick warnings in two samples of Canadian adults who smoke king-size cigarettes, February–August 2024

*Variables*	*Categories*	*Standard survey (unweighted)*				*Daily diary study*	
		*Noticed health information* *on any sticks smoked vs* *none (past month)^a^*		*Noticed health information* *on ≥ almost all sticks* *smoked vs less frequently* *(past month)^a^*		*Noticed information about* *harms on cigarette sticks* *vs other sources or not* *noticing (today)^b^*	
		*%^e^*	*AOR (95% CI)^c^*	*%c*	*AOR (95% CI)^c^*	*%^e^*	*AOR (95% CI)^c^*
**Age** (years)	18–34 ®	73	1	22	1	13	1
	35–49	76	1.60 (0.90–2.84)	42	2.24 (1.23–4.06)*	9	1.04 (0.52–2.07)
	50–64	61	0.82 (0.48–1.40)	38	1.47 (0.83–2.60)	13	1.20 (0.61–2.37)
	≥65	58	0.80 (0.44–1.47)	34	1.24 (0.66–2.32)	12	0.98 (0.47–2.07)
**Sex**	Female ®	63	1	37	1	12	1
	Male	68	1.17 (0.85–1.61)	34	0.89 (0.65–1.23)	12	0.85 (0.59–1.21)
**Race**	White ®	64	1	36	1	11	1
	Other	75	1.33 (0.78–2.27)	36	1.11 (0.66–1.86)	17	1.69 (1.16–2.47)**
**Education level**	High school or lower ®	62	1	33	1	11	1
	Technical school	65	1.02 (0.73–1.43)	38	1.16 (0.81–1.66)	10	1.06 (0.74–1.54)
	College or higher	78	1.62 (0.96–2.74)	40	1.20 (0.74–1.97)	14	0.96 (0.61–1.50)
**Cigarettes per day**	0–9 ®	75	1	40	1	13	1
	10–14	66	0.77 (0.48–1.24)	42	1.04 (0.65–1.66)	13	0.65 (0.43–0.98)*
	15–19	63	0.75 (0.46–1.23)	32	0.91 (0.55–1.51)	10	0.89 (0.58–1.37)
	≥20	60	0.73 (0.47–1.15)	28	0.73 (0.47–1.14)	11	0.89 (0.59–1.36)
**First nation cigarettes in last month**	None ®	79	1	54	1	16	1
	Any	48	0.21 (0.15–0.29)***	9	0.10 (0.07–0.15)***	5	0.60 (0.46–0.79)***
**Roll-your-own use in last month**	None ®	63	1	38	1	12	1
	Any in last 30 days	85	3.92 (2.22–6.92)***	22	0.90 (0.51–1.61)	10	1.11 (0.79–1.56)
**Quit attempt in last 3 months**	None ®	61	1	37	1	12	1
	Any	77	1.59 (1.08–2.33)***	33	0.66 (0.45–0.97)*	12	0.88 (0.68–1.15)
**Intention to quit in next 6 months**	None ®	61	1	34	1	11	1
	In next 6 months	75	1.39 (0.97–1.99)	40	1.74 (1.21–2.49)**	14	1.36 (1.08–1.72)**
**Survey wave**	Feb 2024 ®	NA^d^				7	1
	May 2024 ®	57	1	28	1	11	1.78 (1.29–2.45)***
	Aug 2024	73	2.29 (1.81–2.90)***	43	2.62 (2.03–3.38)***	16	2.90 (1.72–4.90)***
**Time in sample^g^**	Standard (0–4) Daily diary (0–2)	NA^f^	0.95 (0.85–1.07)	NA^f^	1.00 (0.92–1.09)	NA^f^	0.98 (0.77–1.24)

a N=1724 observations in the table from 999 individuals. This item was administered in standard survey.

b N=10572 observations shown in the table from 527 individuals who completed at least 11 out of 14 surveys. This item was administered in daily diary (DD) survey.

c AOR: adjusted odds ratio; adjusted by age, sex, race, education level, cigarettes per day, using any roll-your-own cigarettes, quit attempt, quit intention, purchasing from First Nations Reserve, time in sample.

d Not available because noticing cigarette stick messages was not administered in standard survey, February 2024.

e The percentages are crude.

f Not available because time in sample was treated as continuous in the models.

g Reflects the number of times a participant contributed to the study.

For the standard survey, time-in-sample values ranged from 1–5 (corresponding to participation across waves 5–7 of the original design); in the daily diary, values ranged from 1–3. In the dataset, these ranges are indexed from zero [i.e. standard (0–4), daily diary (0–2)].

* p<0.05.

** p<0.01.

*** p<0.001.

Bolded text indicates statistically significant differences from the reference group, including for crude percentage. ® Reference categories.

None of the models yielded variance inflation factors indicative of issues with collinearity. Furthermore, sensitivity analyses produced consistent results, whether adding weights to the models for the standard survey or including daily diary study participants who contributed fewer than 11 of 14 daily diaries (Supplementary file Figure 1S and Table 1S). The only difference was for the weighted results for the standard survey: one contrast over time was no longer significant (feeling about sticks) and another that was null became significant and positive (thinking about smoking harms).

## DISCUSSION

To evaluate early implementation responses to cigarette stick warnings among adults who smoke in Canada, we used two survey methods. Noticing a change in labeling is likely to be a necessary condition for labeling effects on smoking-related behaviors^18,19^. The fact that some people who smoke actively avoid looking at cigarette pack health warnings^20^ may help explain the lower than expected levels of noticing on-cigarette warnings. However, even avoidance can be associated with desirable cessation outcomes, perhaps because efforts to avoid thinking about something can be indicative of it having an effect (ironic processing)^21-23^. Noticing anti-tobacco information is associated with higher quit intentions and more negative attitudes towards smoking^24^. Both our surveys found increases in noticing cigarette stick warnings over time, indicating that progress on this precursor effect on smoking-related outcomes is being achieved.

The standard survey, but not the daily diary study, found increases in cessation-related responses to stick warnings. Specifically, feeling about cigarette sticks became more negative over the early implementation period. While our measure of feeling about cigarette sticks is new, it was informed by qualitative research exploring response to warnings on cigarette sticks^10^ and is based on theory and measures from studies of the affect heuristic, where feelings are an indicator of perceived risk and predict subsequent decision making^15^. As participants in the standard survey were increasingly exposed to the cigarette stick warnings, they felt worse when looking at their cigarette sticks, which other research using this sample has found to predict subsequent cessation attempts^13^. We also found that standard survey participants reported higher rates of forgoing cigarettes and stronger motivation to quit smoking later in the implementation period. Forgoing of cigarettes is a consistent predictor of making quit attempts^5^, and motivation to quit has predicted quit attempts^25^, cessation, and maintained abstinence^26^. Nevertheless, the changes in all these outcomes were relatively modest, perhaps partly because on-cigarette warnings were implemented in the context of prominent warnings (75% of front and back) on standardized packs. Still, given the broad reach of these messages and the size of the population of people who smoke, this policy may result in meaningful public health outcomes.

These conclusions are tempered by results from the daily diary study, which found no evidence of changes in cessation-related responses to cigarette stick warnings. Our daily diary approach was motivated by the desire to collect data closer to the moments of message exposure and minimize potential recall bias, but this strategy may not have been as successful as we had predicted due to participant burden and demand effects from it. Indeed, the daily diary data from our sample exhibited high ICCs (Figure 1), suggesting that participants’ responses remained relatively constant throughout the collection period. This suggests the possibility that responses were also less thoughtful than desired. A similar concern has been raised in another ecological momentary assessment (EMA) study of cigarette pack labeling that found limited evidence of effects on cessation-related outcomes^27^, whereas the standard approach of analyzing end-of-trial data from the same study data found clearer and more consistent effects^28^. And another study of health warnings on cigarette packages in Australia involving two EMA studies found no immediate increase in outcomes (i.e. self-efficacy, risk appraisal, or quit intention) that were predicted to increase upon exposure to newly implemented pictorial warnings^29^. Further methodological research is needed to determine whether warning effects are better detected when assessed less frequently, as in our standard survey, or whether recall and attribution bias help account for the differences we found. In addition, research is needed to assess long-term impacts of this policy, where standard methods are likely more appropriate and cost efficient.

### Strengths and limitations

Strengths of this study include its use of two different survey approaches involving comparable samples of people who use king-size cigarettes to evaluate the early implementation of on-cigarette warning policy for king-size cigarettes. That this study occurred early in implementation is also a potential limitation, given that it was unclear how many people were exposed to the warnings at any given time; however, such early examination may provide valuable insights on ongoing policy implementation. Additionally, it is unclear how compliant manufacturers or distributors were with the deadlines required the warnings on cigarettes. Confirming the presence of stick warnings may have been impractical for many distributors, given that cigarette packages must be opened for the warnings to be observed. Additionally, although daily data collection is potentially useful for reducing recall issues, the potential of high participant burden and high ICCs in our sample may have prevented us from finding associations in the daily diary study. Finally, information bias and misclassification due to self-report, as well as residual confounding from unmeasured confounders, are potential limitations, though any impact they may have had on the results is not clear.

## CONCLUSIONS

Overall, this study found that adults in Canada who smoke increasingly noticed on-cigarette warnings over the initial policy implementation period, with some evidence of concurrent increases in cessation-related beliefs and behaviors. This suggests that the cigarette stick warning policy in Canada may have had intended, albeit moderate, effects on people who smoke during early policy implementation. Continued monitoring of this policy, which, as of April 2025, is mandatory for all cigarettes in Canada (e.g. including regular size), will be important to determine the long-term effects of this policy.

## Supplementary Material



## Data Availability

The data supporting this research are available from the authors on reasonable request.
