# Supplementary material for: Evaluating early implementation of the innovative Canadian policy of cigarette stick warnings among adults in Canada who smoke: An assessment using repeat cross-sectional surveys and daily diaries
Source: Tob Induc Dis. 2025 Nov 26;23:10.18332/tid/211649. doi: 10.18332/tid/211649 (PMC12649611; doi:10.18332/tid/211649)
Supplement: Supplementary file 1 [file TID-23-185-s1.pdf]

Supplemental Figure 1S. Weighted different trends of response variables among Canadian adults who smoke king size cigarettes in different surveys, February 2024- August 2024

Figure 1.a Trends of noticing cigarette sticks over time among two surveys

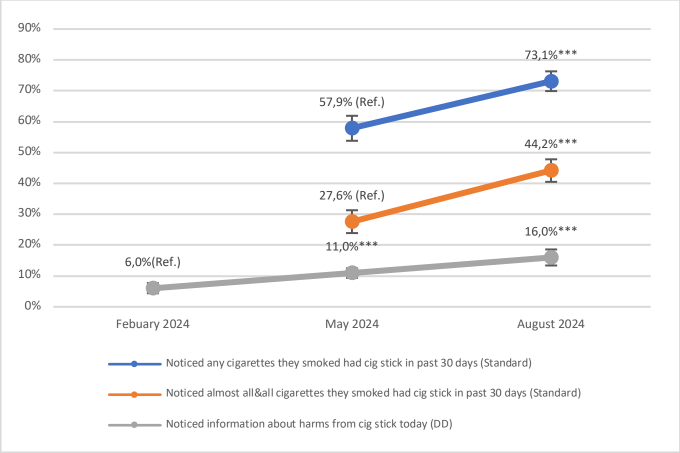

Intra-Class Correlation (ICC) coefficient for daily dairy survey=0.73

Figure 1.b Trends of feeling to cigarette sticks over time among two surveys

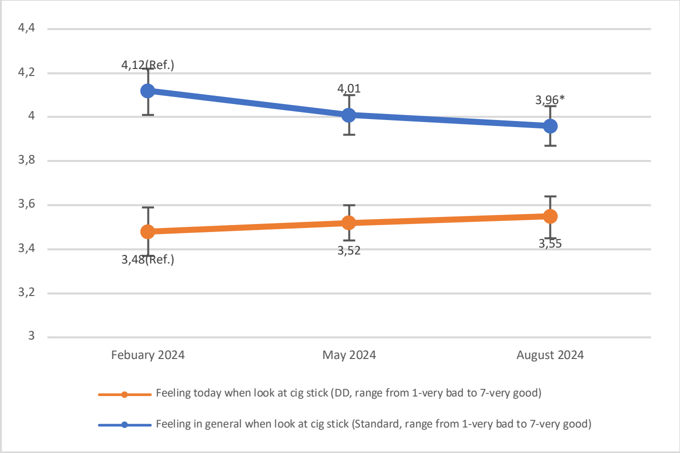

ICC for daily dairy survey=0.67

Figure 1.c Trends of cognitive elaboration over time among two surveys

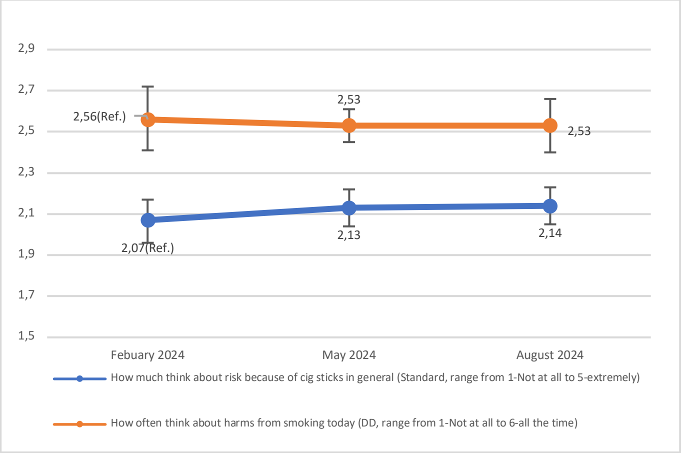

ICC for daily dairy survey=0.74

Figure 1.d Trends of forgoing any cigarettes over time among two surveys

Figure 1.e Trends of motivation to quit smoking over time among two surveys

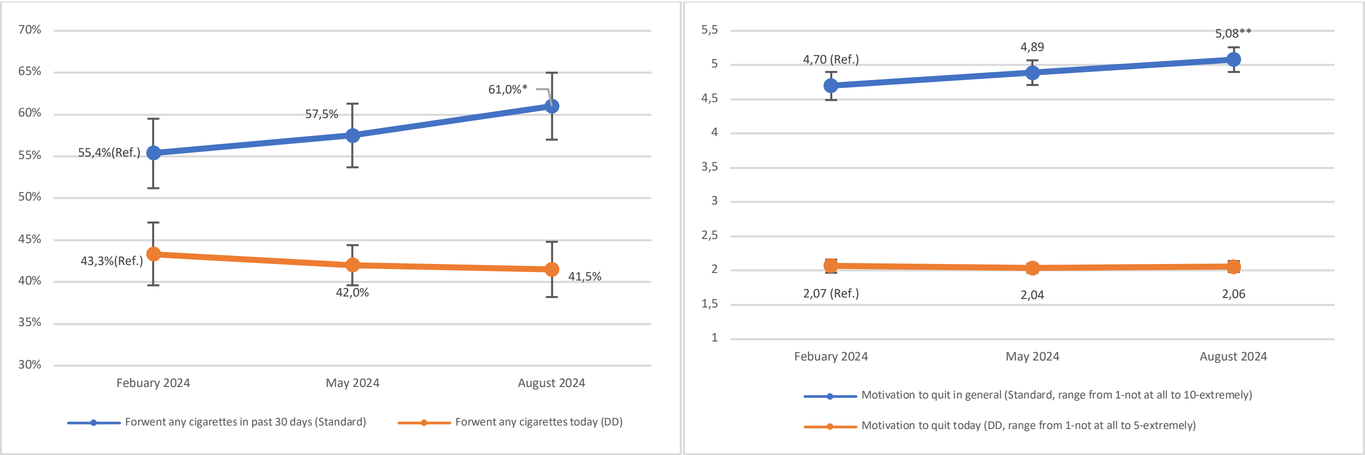

ICC for daily dairy survey=0.78 ICC for daily dairy survey=0.77

Standard survey sample n=1,732 observations in the table from 1,003 individuals. Daily diary sample. n=10,572 observations shown in the table from 527 individuals. The error bars represent confidence intervals. *p<0.05; **p<0.01; ***p<0.001

Supplemental Table 1 Sensitivity Analysis of Correlates of noticing cigarette stick warnings in two samples of Canadian adults who smoke king size cigarettes, February-August 2024

Characteristics		Standard Survey (weighted): Noticed health information on any sticks smoked VS None (past month)[1]		Noticed health information on >=almost all sticks smoked VS less frequently (past month)[1]		Daily Diary Study: Noticed information about harms on cigarette sticks VS other sources or not noticing (today)[2]	
		%[5]	Adjusted OR (95% CI)[3]	%[5]	Adjusted OR (95% CI)[3]	%[5]	Adjusted OR (95% CI)[3]
Age (years)	18-34	73%	Ref.	22%	Ref.	14%	Ref.
	35-49	76%	1.52 (0.82,2.82)	**42%**	**2.25 (1.11,4.58)***	**8%**	**0.59 (0.35,0.98)***
	50-64	61%	0.79 (0.43,1.45)	38%	1.54 (0.77,3.07)	13%	1.09 (0.67,1.76)
	65+	58%	0.77 (0.39,1.49)	34%	1.21 (0.59,2.49)	11%	0.84 (0.49,1.43)
Sex	Female	63%	Ref.	37%	Ref.	11%	Ref.
	Male	68%	1.16 (0.84,1.62)	34%	0.92 (0.66,1.28)	11%	0.86 (0.64,1.15)

Race	White	64%	Ref.	36%	Ref.	11%	Ref.
	Other	75%	1.55 (0.87,2.77)	36%	1.11 (0.61,2.01)	**15%**	**1.60 (1.17,2.20)****
Education	High school or less	62%	Ref.	33%	Ref.	10%	Ref.
	Technical school	65%	1.02 (0.72,1.45)	38%	1.11 (0.77,1.61)	10%	0.96 (0.70,1.31)
	College or more	78%	1.64 (0.95,2.82)	40%	1.26 (0.76,2.10)	14%	1.07 (0.74,1.55)
Cigarettes per day	0-9	75%	Ref.	40%	Ref.	13%	Ref.
	10-14	66%	0.76 (0.46,1.24)	42%	0.93 (0.56,1.53)	**11%**	**0.66 (0.47,0.91)***
	15-19	63%	0.76 (0.45,1.27)	32%	0.89 (0.50,1.57)	9%	0.77 (0.53,1.11)
	>20	60%	0.75 (0.47,1.19)	28%	0.72 (0.45,1.17)	11%	0.85 (0.60,1.19)
First nation cigarettes in last month	None	79%	Ref.	54%	Ref.	16%	Ref.
	Any	48%	**0.18 (0.13,0.25)*****	9%	**0.09 (0.06,0.14)*****	**6%**	**0.61 (0.48,0.77)*****
Roll-your-own use in last month	None	63%	Ref.	38%	Ref.	11%	Ref.
	Any in last 30 days	85%	**4.18 (2.30,7.58)*****	22%	0.89 (0.45,1.76)	11%	1.13 (0.85,1.52)
Quit attempt in last 3 months	None	61%	Ref.	37%	Ref.	11%	Ref.
	Any	77%	**1.49 (1.01,2.20)***	33%	**0.61 (0.41,0.91)***	13%	0.97 (0.78,1.20)
Intention to quit in next 6 months	None	61%	Ref.	34%	Ref.	10%	Ref.
	In next 6 months	75%	**1.55 (1.08,2.23)***	40%	**1.81 (1.25,2.63)****	**14%**	**1.37 (1.12,1.67)****
Survey wave	Feb 2024	NA[4]				7%	Ref.
	May 2024	57%	Ref.	28%	Ref.	12%	**1.98 (1.55,2.54)*****
	Aug 2024	73%	**2.35 (1.84,3.02)*****	43%	**2.58 (1.97,3.39)*****	**16%**	**3.08 (2.09,4.54)*****
Time in sample	Standard [0-4]<br>Daily diary [0-2]	NA[6]	0.95 (0.84,1.08)	NA[6]	1.10 (0.98,1.24)	**NA[6]**	0.94 (0.78,1.12)

1. n=1,724 observations in the table from 999 individuals. This item was administered in standard survey
2. n=10,572 observations shown in the table from 527 individuals who completed any of the surveys. This item was administered in daily diary (DD) survey
3. CI- Confidence interval. Adjusted by age, sex, race, education, cigarettes per day, using any roll-your-own cigarettes, quit attempt, quit intention, purchasing from First Nations Reserve, time in sample
4. Not available because noticing cigarette stick messages was not administered in standard survey, February 2024
5. The percentages are crude

6. Not available because time in sample was treated as continuous in the models

*: <0.05; **:<0.01; ***:<0.001 Bolded text indicates statistically significant differences from the reference group, including for crude percentage.

© **2025 Hackworth E.E. et al.**
